# Workplace violence against mental health professionals in Italy: a nationwide survey on prevalence and risk perception

**DOI:** 10.1007/s00406-026-02197-y

**Published:** 2026-03-13

**Authors:** N. Granata, C. Gesi, A. Fagiolini, G. Migliarese, M. Rocchetti, G. Cerveri

**Affiliations:** 1 Department of Mental Health and Addiction, ASST Lodi, Lodi, Italy; 2 Department of Mental Health and Addiction, ASST Fatebenefratelli-Sacco, Via GB Grassi 74, 20157 Milan, Italy; 3 Department of Molecular Medicine, Psychiatric Division, University of Siena, Siena, Italy; 4 Department of Mental Health and Addiction, ASST Pavia, Pavia, Italy; 5 Department of Brain and Behavioral Sciences, University of Pavia, Pavia, Italy

**Keywords:** Mental health workers, Violence, Risk perception, Survey

## Abstract

Workplace violence in healthcare, particularly within mental health settings, is a growing global concern, associated with significant impairment on professionals’ well-being and working performance. This survey aimed to analyze the incidence of physical and verbal aggression against Mental Health Workers (MHWs) across Italy and its impact on their perceived sense of security. A total of 2704 MHWs participated in an online survey conducted in May 2023. The results revealed that 45.9% of respondents had experienced physical aggression in the previous two y.ears, with 26% being assaulted multiple times. 67.8% reported verbal threats in the last three months, multiple times in 46.9% of the sample. Inpatient psychiatric services were identified as the highest-risk environments for violence, with psychiatrists and nurses being the most affected professional groups. The study also found that male MHWs experienced higher rates of physical aggression, while older age slightly reduced the risk of violence. Finally, previous exposure to violence strongly correlated with increased feelings of insecurity at work. The findings highlight the urgent need for systemic interventions, including improved safety protocols, design for safer workplace environments, continuous training, and mental health support to mitigate the psychological and professional burden on MHWs, and ensure a safer, more supportive working environment.

**Supplementary Information:**

The online version contains supplementary material available at 10.1007/s00406-026-02197-y.

## Introduction

 Workplace violence is defined as “incidents where personnel are abused, threatened, or attacked in circumstances related to their work, including commuting to and from work, which implies an explicit or implicit challenge to their safety, well-being, or health” [1]. This term encompasses not only physical aggression but also situations in which personnel are subjected to sexual, verbal, and non-verbal harassment or intimidation [2]. Workplace violence is increasingly documented all over the world, being one of the most serious issues affecting the healthcare sector [3, 4]. A recent global survey collecting data from 79 countries (5405 subjects) revealed that 55% of healthcare professionals interviewed had experienced at least one episode of violence in the previous year, the most common form being verbal (50.9%), followed by emotional (29.5%) and physical (21.4%) [5]. In Italy, the National Institute for Insurance against Accidents at Work (INAIL) reported approximately 6000 aggressions against health workers from 2020 to 2022, with 2243 events occurring only in 2022, and corresponding to a 14% increase compared to the previous year [6]. Although violence against workers represents an urgent concern that spans across the entire field of healthcare, existent literature highlights that Mental Health Workers (MHWs) might be especially prone to violence on the workplace and to consequent psychological distress, from work exhaustion to burnout, sleep disorders, depression, and PTSD [7–13]. As a matter of fact, a recent umbrella review that considered 32 systematic reviews, including 19 with meta-analyses, the prevalence of violent acts against healthcare workers was about 80%, with a particularly significant impact on nurses working in mental health settings [10]. A recent systematic review of studies involving MHWs from US inpatient services showed prevalence estimates of physical aggression in the previous year ranging between 25 and 85%. In surveys conducted across several different countries, the prevalence of violence on MHWs in the previous year ranged between 44% and 95% [14–18]. The great variability among studies is arguably due to several factors, including both methodological and cultural factors. Moreover, the majority of extant studies comes from psychiatric wards, a minority from forensic psychiatric services and only few studies, eventually, evaluated violence across a broader range of psychiatric settings and professional roles. Among these, a recent Italian study assessed violence in a mixed sample of 183 MHWs from psychiatric wards, community outpatients services and university hospitals from Northern and Southern Italy, reporting an overall 90% prevalence of verbal threat in the lifetime, and 45% in the previous year. Moreover, about 50% had been physically attacked in their lifetime while the same figure dropped to about 5% in the previous year. Overall, psychiatrists had been exposed to violence to a greater degree than nurses, rehabilitation personnel and psychologists both in their lifetime and in the previous year [19]. A later study conducted among 164 MHWs from a psychiatric department in Northern Italy reported a 91.5% prevalence of aggression, a 45% of physical attack and a 24% of injury in the previous year, with female professionals, working in inpatient settings, and having less work experience identified as significant predictors of physical aggression [20]. Two other Italian studies had previously focused only on psychiatrists. In 2010 Catanesi et al. reported that among 1202 Italian psychiatrists, almost all (91%) had experienced verbal aggression in their work life; 72% had been threatened with dangerous objects and 65% had suffered physical aggression, with inpatient care units being the work environment at higher risk [21]. A later study from the same group conducted among psychiatrists from Apulia region, in Southern Italy, found about a 67% lifetime prevalence of verbal violence, a 66% of physical violence and a 38% of stalking, with female psychiatrists showing a higher risk of workplace violence, particularly verbal abuse, and greater psychological consequences after suffering verbal abuse [22]. Despite the above studies suggesting the need of careful consideration of violent behaviors toward MHWs, current literature lacks of nationally representative, comprehensive samples of MHWs, covering the whole spectrum of professional roles and settings of mental care. Following a recent case of workplace violence, which resulted in the killing of an Italian psychiatrist, several Italian psychiatrists and other mental health professionals, gathered in several web-based groups, aiming to share their experiences of violence on the workplace and to collect proposals to face this issue. The Italian Psychiatric Association (SIP) thus promoted the present survey to collect data on the prevalence and risk perception of violence and associations with age, gender, professional role, and work setting in a nationally representative sample of MHWs. Results will contribute to both improve current literature and set the ground for intervention within the national healthcare system in the field of mental health.

## Methods

### Participants and materials

This online survey collected data from a national sample of Italian Mental Health Workers (MHWs), From May 2nd to May 12th 2023, MHWs were invited to participate in the study through mailing lists, social media, and snowball sampling. Participants who agreed to take part in the study were invited to complete an online informed consent form, which ensured anonymity and authorized the use of their data for research purposes. Upon providing consent, participants received a link to an online questionnaire. MHWs were enrolled independently from age, gender, department and job title, the only exclusion criterion being refusal to sign the informed consent form. The survey was meant to health professionals only. Patients, patient care, or personal health data were not involved. Since the activity was strictly limited to gathering professional perspectives and did not involve patient data or issues pertaining to patients or professionals’ health, formal approval from an ethics committee or an Institutional Review Board was not sought. The online questionnaire was specifically designed by the authors to be concise (with an estimated completion time of approximately 10 min) while comprehensively addressing different thematic areas about violence against MHWs. The first part was designed to gather information about the age, gender, profession, and work environment of the participants. The second part comprised 13 close-ended questions, each with three response options based on the frequency of an event or the level of agreement with the content of the question. The questions were grouped into four thematic categories based on their content: *perception of risk* (3 questions, see Appendix 1), *responsibility in clinical practice* (5 questions), *definition of intervention protocols* (3 questions), and *improvement in work quality* (2 questions). Two open-ended questions were added at the end of the questionnaire to allow people suggestions or if there were any aspects not considered in the previous questions.

In the present report, we focused on analyzing the first three questions, which address the perception of risk among MHWs.

### Statistical analysis

Descriptive statistics including numbers and percentage for categorical variables were provided for the primary aim. As secondary analyses, logistic regressions were conducted to investigate predictors of physical aggression, threats and risk perception among MHWs. To be included as dependent variable in the first regression, the variable “physical aggression” was dichotomized as having been assaulted or not, in the previous two years. For the second regression, the variable “threat” was dichotomized as having been threatened or not in the previous three months. For the third logistic regression, the variable “perception of risk” was dichotomized as feeling at risk at least most of the time, or feeling at risk sometime or never while at work. Bonferroni correction was applied to safeguard against Type 1 error due to multiple comparisons (level of significance was set at two tailed corrected p-value = 0.003).All analyses were conducted with SPSS version 29.0.2 [23].

## Results

The total sample included 2704 MHWs, mean age 46.91 (± 11.13), 807 males (29.8%), and 1897 females (70.2%). Whit regard to professional role, 1401 (51.8%) were psychiatrists, 561 (20.7%) nurses, 198 (7.3%) psychologists, 154 (5.4%) rehabilitation personnel, 212 (7.8%) other professionals (mainly including nurse auxiliary personnel, social workers and volunteers) and 178 (6.6%) other medical doctors. 1285 (47.5%) worked in the Community Outpatient Services (COS), 590 (21.8%) in Inpatient Services (IS), 314 (11.6%) in Long-Term Mental Health Facilities (LMHF), 126 (4.7%) in University Hospitals (UH), 68 (2.5%) in Forensic Mental Health Services (FMHS), and 321 (11.9%) in other services. The extended data are showed in Table 1.

1463 (54.1%) subjects reported no physical aggression in the previous two years, 537 (19.9%) reported being assaulted once, and 704 (26.0%) two or more times. In the previous three months, 899 (33.2%) reported no threats, 536 (19.8%) reported being threatened once, 1269 (46.9%) twice or more times. 1446 MHWs (53.5%) stated they felt at risk most of the time or always while at work, 1258 (46.5%) sometime or never.

### Predictors of physical aggression and threats

Two logistic regression models were conducted using physical aggression in the previous two years and threats in the previous three months as respective dependent variables, and age, sex, type of service and work role as independent ones. Results are shown in Table 2. Female sex was shown to decrease the risk of physical aggression [OR = 0.73 (0.61-0.87)], while older age was shown to marginally decrease the risk of both [OR = 0.98 (0.98-0.99) for physical aggression OR = 0.99 (0.98-0.99) for threats]. As for the workplace, assuming the COS as reference category, working in the IS was found to significantly increase the risk of both physical aggression [OR = 2.86 (2.31–3.54)] and threats [OR = 3.39 (2.59–4.44)]. In addition, working in FMHS significantly increased the risk of being threatened [OR = 2.69 (1.41–5.18)]. All professional roles were shown to have a significant lower risk of Threats in the previous three months compared to Psychiatrists (ORs from 0.20 to 0.63). All professional roles but nurses were shown to have a significant lower risk of aggression in the previous three years compared to Psychiatrists (ORs from 0.29 to 0.66).

### Predictors of feelings at risk on the workplace

A third logistic regression was conducted using the feeling of insecurity at work (i.e. feeling at risk most of the time or always vs. feeling at risk sometime or never) as dependent variable, and age, sex, type of service, work role, history of physical aggression in the previous two years and history of threats in the previous three months as independent ones (Table 2). The latter was shown to significantly increase the odd of feeling at risk most of the time/always [OR = 4.24 (3.50–5.16)]. Aggression(s) in the previous two years also strongly predicted feelings of insecurity [OR = 2.87 (2.40–3.44)]. Further predictors that significantly increased the odd of feeling at risk were female sex (OR = 1.70), and working in the IS (OR = 1.62 compared to working in the COS). Psychologist and Rehabilitation personnel roles (OR = 0.53 and OR = 0.68 compared to Psychiatrist role) and working in LMHF or Other [OR = 0.61 (0.45-0.83) and OR = 0.54 (0.40-0.73) respectively] were shown to significantly decrease the odd for feeling at risk on the workplace compared to working in the COS.

## Discussion

With this study, we aimed at providing a nation-wide picture of the risk of violence on the workplace among MHWs in Italy. By means of an online survey, we gathered data from an overall sample of 2704 MHWs, 2483 of whom working in the Public Health System (PHS). As the national MHWs workforce was estimated at 40285 professionals in the 2022, 30101 of whom working in the PHS [24], our sample accounts for about 6.7% of the overall MHWs workforce and 8.3% of the MHWs working in the Italian PHS respectively. Results revealed that more than a half of the participants had been verbally threatened in the three months before participating in the survey, and nearly a half had experienced physical aggression in the previous two years. Further, more than half of MHWs enrolled reported to feel at risk for most of their time at work.

Regarding socio-demographic variables, female sex was found to significantly decrease the risk of physical assault. Our findings partially align with some previous studies on MHWs, reporting that females are more likely to experience verbal threats, while males are more likely to be physically attacked [25–28]. Still, another body of studies failed to find a difference between males and female [14, 29–32]. Findings regarding gender are controversial; however, in our study, consistent with the literature, males were found to be at higher risk of physical aggression. Older age was also found to slightly reduce the odd of aggression and threat, suggesting that professional experience may play a protective role toward violent behaviors on the workplace [33]. Regarding the professional role, the odd of both physical and verbal threats were significantly lower for all professionals but nurses, compared to psychiatrists. This observation is in agreement with a large study involving 10821 healthcare workers, where physicians and nurses reported the highest percentages of workplace violence, with 62.3% and 20%, respectively [34]. Furthermore, a scoping review of studies conducted in Italy revealed a high prevalence of workplace violence against healthcare workers, with nurses and physicians being the most affected, particularly in psychiatric and emergency departments [13]. These findings suggest that psychiatrists and nurses, who have direct and frequent contact with patients, especially in crisis situations, are at greater risk of encountering aggression in mental health settings. This statement is further corroborated by a recent literature review, which indicates that workplace violence is more prevalent in psychiatric departments compared to other wards, such as emergency services, polyclinics/waiting rooms, and geriatric units [35]. Somewhat expectedly, our data also revealed that professionals working in the IS have a significantly higher risk of both threat and physical violence in comparison to professionals working in the COS. Arguably, patients treated in the IS may present with emergency or unstable mental states, contributing to the greater likelihood of violent behaviors. Interestingly, also MHWs from FMHS present greater risk of being threatened, but not assaulted, compared to those from the COS. As a whole, IS and SMHF stick out as the work environments bearing the higher risk of violence for MHWs [36, 37]. As such, they should be given special attention when it comes to implement strategies to prevent violent behaviors. According to our data, these strategies could also include a thoughtful distribution between male and female professionals and across seniority levels. As a further aim, we investigated the risk perception of MHWs. Results revealed that the risk perception is significantly higher among female professionals, compared to males, and among other medical figures compared to psychiatrists. On the contrary, psychologists’ and rehabilitation personnel’s risk perception is almost a half of psychiatrists’ perceived risk. The greater risk perception among females might arguably depend on females being more often threatened as well as on their feeling of physical disparity when dealing with male patients on the workplace. Moreover, the higher proneness to psychological sequelae after traumatic experiences on the workplace among females and their impact on functioning may also play a role [38, 39]. As for the higher risk perception among other medical doctors compared to the reference category of psychiatrists, we might argue that the unfulfilled psychiatric training of both residents and medical doctors working in the Addiction services within this category might increase the perception of risk. Expectedly, having been threatened in the previous three months and having been assaulted in the previous two years were the strongest predictors of risk perception. These findings highlight the importance of preventing violence on the workplace also as a safeguard for MHWs’ mental health, quality of life, and job satisfaction. In proceeding with various studies, in fact, workplace violence can significantly affect subjective well-being, and contribute to the development of burnout and post-traumatic stress disorder (PTSD) among MHWs [8, 19, 40]. The emotional burden associated with feeling unsafe at work may not only diminish job performance but also hinder the therapeutic relationship between healthcare providers and patients, further exacerbating challenges in mental health care settings [19]. The study presents both strengths and limitations. As to limitations, an imbalance was observed in the percentage of psychiatrists compared with other professional groups, which may be related to the survey dissemination process. Since the initiative was promoted by the Italian Society of Psychiatry (SIP), psychiatrists were primarily contacted and subsequently shared the questionnaire with other MHWs, which may have contributed to the role-related selection bias. Another limitation is the lack of regional data, as the survey was designed to collect only nationally homogeneous information. Finally, the survey did not include a deeper representation of psychological distress. While this choice was made in order to keep the survey brief and to avoid sensitive health data, it would be advisable in future studies to include short, standardized, and widely used assessment tools for anxiety, depression, and post-traumatic stress symptoms. In the context of the above limitations, the study provides a broad sample of MHWs, which may contribute to fill a gap in the Italian context, where, to the authors’ knowledge, similar studies are lacking. The comprehensive nature of the data gathered offers an important contribution to understanding the challenges faced by MHWs in Italy. Future studies might profitably investigate region-specific data, as well as focus on comparing rates of violence among MHWs to those found among other healthcare professionals. A further way forward might be to conduct stratified analyses combining demographic, professional, and contextual variables to inform tailored interventions.

## Conclusions

Workplace violence is a critical issue in healthcare, particularly in mental health settings. The study reveals that more than half of professionals have experienced verbal threats, and nearly half have faced physical aggression, with differences by gender, as more physical attacks were suffered by men. Psychiatrists and nurses, especially in inpatient psychiatric services, are at the highest risk. As violence may negatively impact mental well-being, job satisfaction, and the quality of care, careful monitoring of violence in psychiatric settings and the implementation of targeted interventions to improve safety and support for MHWs are both urgently needed. Future strategies could include providing healthcare facilities with adequate spaces for managing critical situations, continuous training programs for MHWs, and the enrollment of security personnel dedicated to ensuring staff safety.

**Table 1:** Participants’ socio-demographic and professional data (*n* = 2704, mean age 46.91 ± 11.13)

	*n* (%)
Gender	
Female	1897 (70.16)
Male	807 (29.84)
Professional role	
Psychiatrist	1401 (51.8)
Nurse	561 (20.7)
Psychologist	198 (7.3)
Rehabilitation personnel	154 (5.7)
Other medical doctors*	178 (6.6)
Other professional figure	212 (7.8)
Work place	
Community Outpatient Service	1285 (47.5)
Inpatient Service	590 (21.8)
Long-term Mental Health Facility	314 (11.6)
University hospital	126 (4.7)
Forensic Mental Health Service	68 (2.5)
Other°	321 (11.9)

*residents, child and adolescent psychiatrists; medical doctors non-psychiatrist within addiction services

°addiction outpatient service; private inpatient service; private psychiatric outpatient practice

**Table 2:** Results of three individual logistic regression analyses for predictors of *Physical aggression*, *Threats* and *Risk perception*

	Physical aggression in the previous two years OR (95% CI)	Threats in the previous three months OR (95% CI)	Feeling at risk most of the time/always OR (95% CI)
Age	0.98 (0.98-0.99)^*^	0.99 (0.98-0.99)^*^	1.00 (0.99 − 1.00)
Gender			
male	1 [reference]	1 [reference]	1 [reference]
female	0.73 (0.61-0.87)^*^	1.24 (1.03–1.50)	1.70 (1.40–2.07)^*^
Professional role			
Psychiatrist	1 [reference]	1 [reference]	1 [reference]
Nurse	1.08 (0.88-1.33)	0.64 (0.51-0.80)^*^	0.87 (0.58 − 1.30)
Psychologist	0.29 (0.19-0.42)^*^	0.20 (0.14-0.28)^*^	0.53 (0.36-0.78)^*^
Rehabilitation personnel	0.66 (0.45-0.97)	0.40 (0.28-0.58)^*^	0.68 (0.47-0.99)
Other medical doctors	1.08 (0.88-1.33)	0.63 (0.50-0.80)^*^	1.30 (1.02–1.65)
Other professionals	0.62 (0.45-0.85)^*^	0.46 (0.34-0.64)^*^	0.97 (0.68-1.36)
Workplace			
Community Outpatient Service	1 [reference]	1 [reference]	1 [reference]
Inpatient Service	2.86 (2.31–3.54)^*^	3.39 (2.59–4.44)^*^	1.62 (1.27–2.07)^*^
Long-term Mental Health Facility	1.10 (0.83-1.46)	0.96 (0.73-1.27)	0.61 (0.45-0.83)^*^
University Hospital	1.42 (0.96-2.13)	0.97 (0.64-1.47)	0.97 (0.62 − 1.50)
Forensic Mental Health Service	1.63 (0.99-2.69)	2.69 (1.41–5.18)^*^	1.22 (0.69-2.15)
Other	0.96 (0.73-1.25)	0.78 (0.60-1.01)	0.54 (0.40-0.73)^*^
Aggression in the previous 2 years^a^	-	-	2.87 (2.40–3.44)^*^
Threat in the previous three months^a^	-	-	4.24 (3.50–5.16)^*^

^*^significant at 2-sided *p* < .003 (Bonferroni-adjusted p-value)

^a^ one or more

## Supplementary Information

Below is the link to the electronic supplementary material.

Supplementary Material 1

## Appendix

See Table 3

**Table 3:** Survey’s closed-ended questions

Questions
Perception of risk
Have you experienced assaults, or violent acts (not verbal threats) in the last two years?
Have you experienced verbal threats in the last three months?
Do you feel at risk for your safety in your workplace?
