# Supplementary material for: Workplace violence against mental health professionals in Italy: a nationwide survey on prevalence and risk perception
Source: Eur Arch Psychiatry Clin Neurosci. 2026 Mar 13;276(4):1725–31. doi: 10.1007/s00406-026-02197-y (PMC13233875; doi:10.1007/s00406-026-02197-y)
Supplement: Supplementary file 1 — Supplementary Material 1 [file 406_2026_2197_MOESM1_ESM.docx]

**Appendix 1.** Survey’s closed-ended questions

| **Questions** |
| --- |
| **Perception of risk** |
| - Have you experienced assaults, or violent acts (not verbal threats) in the last two years? |
| - Have you experienced verbal threats in the last three months? |
| - Do you feel at risk for your safety in your workplace? |
